# Blocking late stages of splicing quickly limits pre-spliceosome assembly in vivo

**DOI:** 10.1080/15476286.2019.1657788

**Published:** 2019-09-04

**Authors:** Gonzalo I. Mendoza-Ochoa, J. David Barrass, Isabella E. Maudlin, Jean D. Beggs

**Affiliations:** a Wellcome Centre for Cell Biology, School of Biological Sciences, University of Edinburgh, Edinburgh, UK

**Keywords:** Auxin, pre-mRNA splicing, Prp22, protein depletion, yeast

## Abstract

Pre-messenger RNA splicing involves multi-step assembly of the large spliceosome complexes that catalyse the two consecutive trans-esterification reactions, resulting in intron removal. There is evidence that proof-reading mechanisms monitor the fidelity of this complex process. Transcripts that fail these fidelity tests are thought to be directed to degradation pathways, permitting the splicing factors to be recycled. While studying the roles of splicing factors in vivo, in budding yeast, we performed targeted depletion of individual proteins, and analysed the effect on co-transcriptional spliceosome assembly and splicing efficiency. Unexpectedly, depleting factors such as Prp16 or Prp22, that are known to function at the second catalytic step or later in the splicing pathway, resulted in a defect in the first step of splicing, and accumulation of arrested spliceosomes. Through a kinetic analysis of newly synthesized RNA, we observed that a second step splicing defect (the primary defect) was rapidly followed by the first step of splicing defect. Our results show that knocking down a splicing factor can quickly lead to a recycling defect with splicing factors sequestered in stalled complexes, thereby limiting new rounds of splicing. We demonstrate that this ‘feed-back’ effect can be minimized by depleting the target protein more gradually or only partially, allowing a better separation between primary and secondary effects. Our findings indicate that splicing surveillance mechanisms may not always cope with spliceosome assembly defects, and suggest that work involving knock-down of splicing factors or components of other large complexes should be carefully monitored to avoid potentially misleading conclusions.

## Introduction

**Figure 1.**
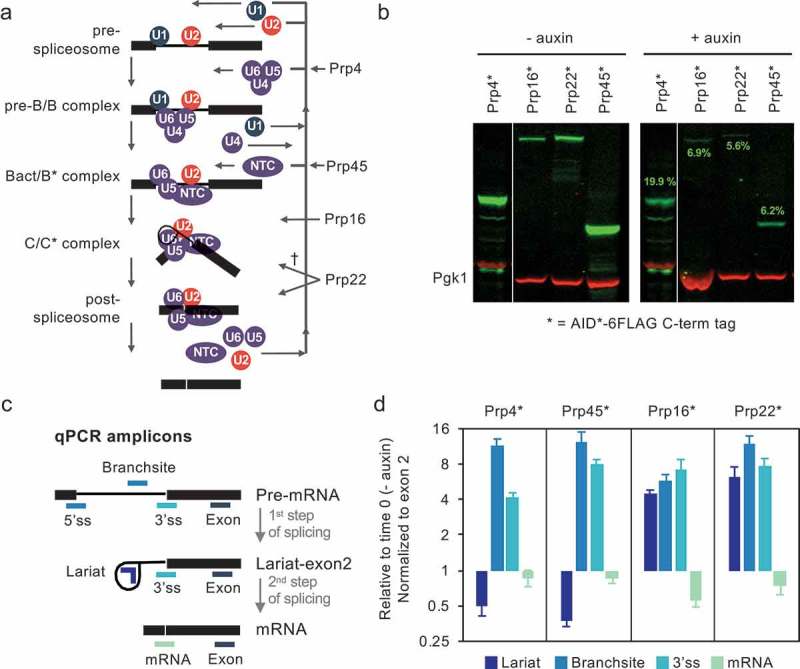
Depletion of splicing factors Prp4, Prp22, Prp16 or Prp45 leads to a first step of splicing defect. (a) Spliceosome assembly model showing recruitment of U1, U2, U4, U5 and U6 snRNPs, nineteen complex (NTC) and splicing factors that were depleted in this study, and their critical stage of activity. (b) Auxin-dependent targeted depletion assessed by immunoblotting with anti-FLAG and anti-Pgk1 (internal control) antibodies. Prp4 was depleted in strain PADH1-409-TIR1. Prp16, Prp22 and Prp45 were depleted in strain PADH1-701-TIR1. Target protein levels after depletion (+ auxin) are shown in green numbers as a percentage of the starting amount, and represent the average of three biological replicates. The anti-FLAG signal was normalized against Pgk1, which is encoded by an intronless gene and was used as internal control. Only one representative blot is shown. (c) Illustration of qPCR-amplified regions from *ACT1* and *ECM33* transcript: 5ʹ splice site (5’ss) (*ECM33* only), Branchsite (BS) (*ACT1* only), 3ʹ splice site (3’ss), exon 2, lariat and spliced mRNA. 5’ss and BS primers only detect pre-mRNAs, 3’ss primers will detect pre-mRNA and also lariat-exon2, a product of the first step of splicing. Lariat will detect both the lariat-exon2 and excised lariat (a product of the second step of splicing). mRNA is produced in the second step of splicing. Exon primers are used as controls to normalize for transcription. (d) qPCR of splicing intermediates of *ACT1* at 30 min depletion, normalized to exon 2 and relative to no depletion (time 0). Pre-mRNA accumulation (increase 3´ss and BS) is indicative of a first step of splicing defect. Error bars denote standard error of biological triplicates. † a second step function of Prp22 may not be required for splicing of all intron-containing transcripts[20].

Pre-messenger RNA (pre-mRNA) splicing is the process by which introns are removed from RNA transcripts and the coding sequences are joined by two consecutive trans-esterification reactions catalysed by the spliceosome (reviewed in [1–3]). The spliceosome is a multi-megadalton RNA-protein complex that is assembled from five small nuclear ribonuclear protein particles (snRNPs) (U1, U2, U4, U5 and U6 snRNPs) plus non-snRNP proteins, including the nineteen complex (NTC) and NTC-related proteins. Spliceosome assembly is a highly dynamic process (Fig. 1a). Briefly, U1 and U2 snRNPs recognize and bind at the intron 5ʹ splice site (5’ss) and branch site (BS), respectively, to form the pre-spliceosome, or A complex. Association of the U4/U6.U5 triple snRNP produces a transient pre-B complex from which the U1 snRNP is displaced to produce the more stable B complex, then removal of the U4 snRNP and recruitment of NTC forms the Bact complex, followed by a further reorganization to create the catalytically active B* complex. The first catalytic step of splicing takes place, and the resulting C complex is remodelled again (C* complex), leading to the second catalytic step. Finally, the post-catalytic spliceosome is actively disassembled and the components are recycled.

Thanks to extensive biochemical and genetic studies (reviewed in [4]), together with high-resolution structures obtained by cryo-electron microscopy [5–13], we now have a comprehensive mechanistic understanding of splicing. However, until relatively recently, pre-mRNA splicing was studied mainly as an isolated process whereas, within the context of the cell, splicing functionally interacts with other cellular systems such as transcription, chromatin and RNA processing (reviewed in [14]).

Most splicing factors are essential for viability. Therefore, in vivo studies of the roles of pre-mRNA splicing factors have generally involved the use of conditional mutants (in yeast) or targeted knock-down of individual factors (e.g. by RNAi in higher eukaryotes). In this study, we were particularly interested in Prp16 and Prp22, which are members of the family of DEAH-box RNA-stimulated ATPases, or RNA helicases, that promote structural rearrangements in splicing complexes (reviewed in [15]). Prp16 binds at, or near, the 3’ss, and triggers the formation of C complex and activation of the catalytic core [5,6,16], whereas Prp22 sits downstream of the 3’ss and releases the spliced mRNA from the post-spliceosome [5,17–19]. Prp22 has also been implicated in the second catalytic step of splicing [20] and in 3’ss selection [21,22].

Our goal was to study the effects on splicing efficiency and co-transcriptional spliceosome assembly of knocking down Prp16 or Prp22 in vivo. For comparison, we studied two other splicing factors, tri-snRNP protein Prp4 and NTC-related protein Prp45, that are involved in different stages of spliceosome assembly. To achieve a fast and specific depletion of our target proteins we used the auxin-inducible degron system [23,24].

Although surveillance mechanisms have the ability to identify defective splicing complexes and target them for dissociation and recycling of the components, our results show that rapid depletion of a splicing factor in vivo can limit the early steps of spliceosome assembly, indicating that the recycling process is overwhelmed. Consequently, the observed phenotypes do not reflect the primary function of the depleted factor. In the case of Prp22, we demonstrate that a more gradual and less complete depletion strategy allows for separation of the primary and secondary effects. We conclude that the budding yeast surveillance and recycling processes cannot cope with large-scale inhibition of splicing, highlighting the need for caution in interpreting the results of in vivo knock-down studies to analyse the roles of different components of a biochemical pathway.

## Methods

### Yeast strains and growth conditions

**Figure 2.**
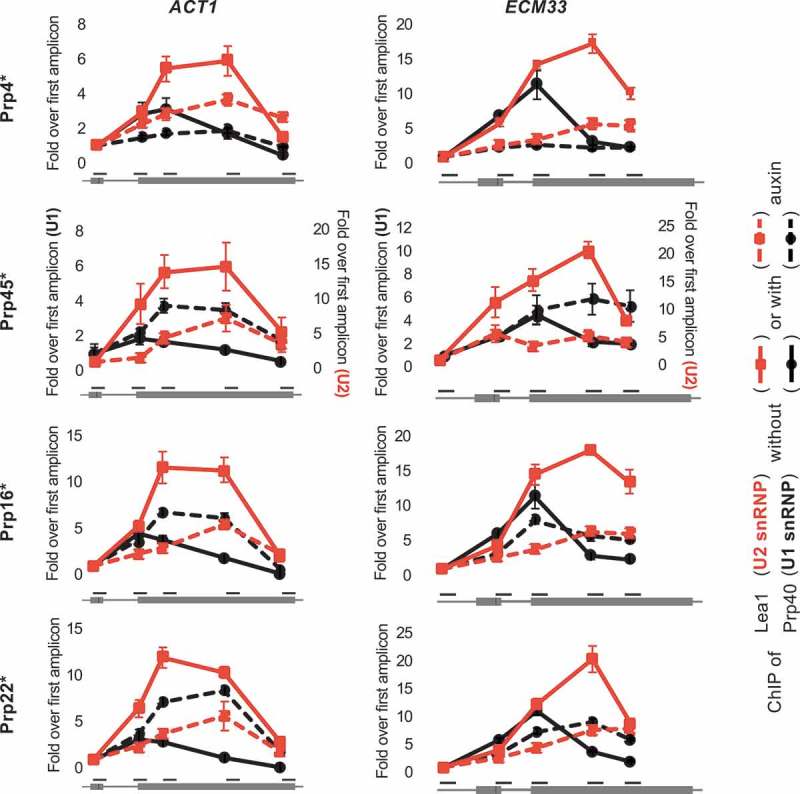
Depletion of several splicing factors (as in Fig. 1) leads to reduced co-transcriptional recruitment of Lea1 (U2 snRNP). ChIP of Prp40 (U1 snRNP; black lines) and HA-tagged Lea1 (U2 snRNP; red lines) on *ACT1* (left plots) and *ECM33* (right plots) genes before (solid lines) and after (dashed lines) auxin-induced depletion. The x-axis represents amplicon location within the gene. For each protein, the ChIP data are presented as relative to the first amplicon for that protein – in the exon 1 of *ACT1* and 5ʹUTR of *ECM33*. Error bars denote standard error of biological triplicates. U1 and U2 ChIP values following Prp45 depletion are at different scales (y-axis values of U1 ChIP are shown on the left of the graph and U2 ChIP values on the right) due to experimental variations (e.g. variations in immunoprecipitation efficiencies). * = AID*-6FLAG C-terminus tag.

**Figure 3.**
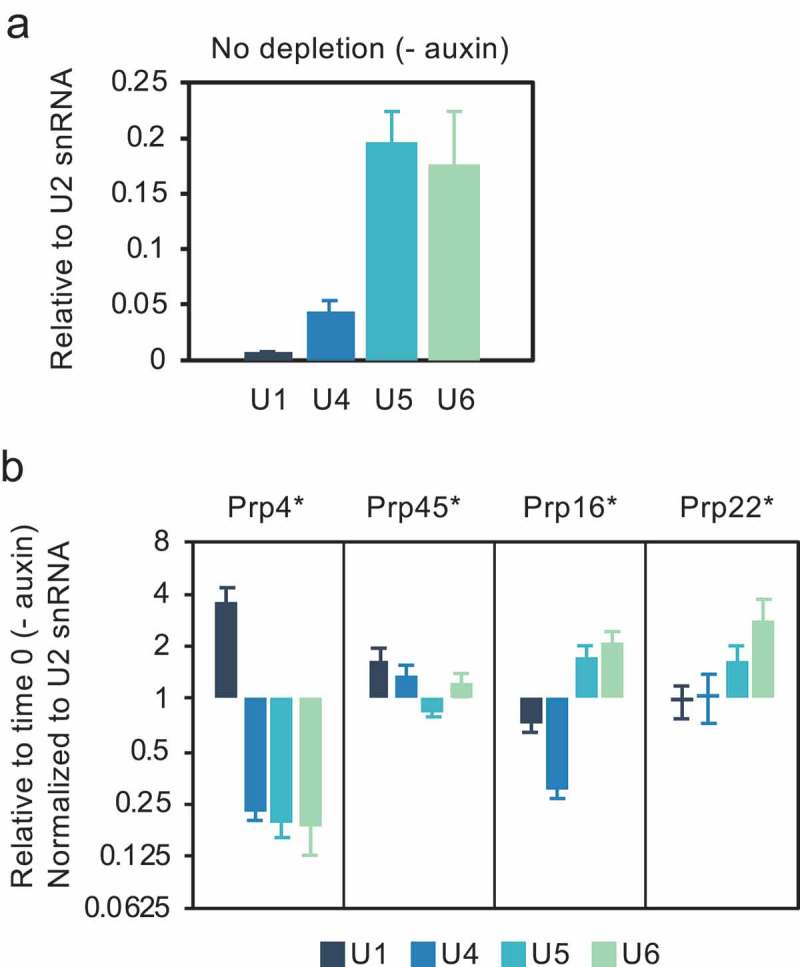
Level of association of U2 snRNP with U1, U4, U5 and U6 snRNAs is altered in the absence of certain splicing factors. (a) RT-qPCR measurement of snRNAs associated with immunoprecipitated, HA-tagged Lea1, a core component of U2 snRNP. (b) RNA immunoprecipitation (RIP) analysis as in panel a after depletion of Prp4, Prp45, Prp16 or Prp22 as in Fig.1. Data are normalized to U2 snRNA signal and presented as relative to no depletion (time 0). Error bars denote standard error of biological triplicates. * = AID*-6FLAG C-terminal tag.

**Figure 4.**
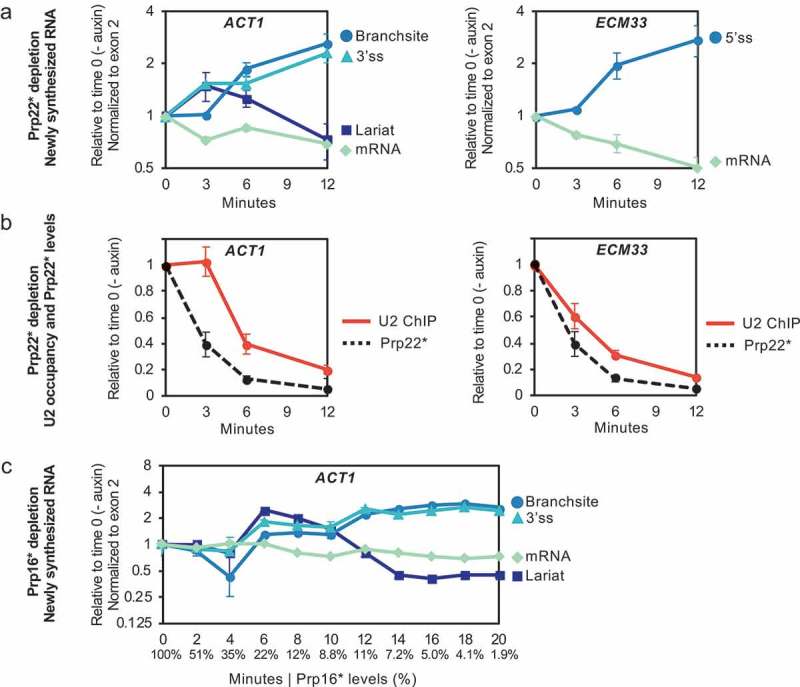
Kinetic analysis of newly synthesized RNA after depletion of Prp22 or Prp16. (a) RT-qPCR analysis of 4tU-labelled newly synthesized RNA of *ACT1* and *ECM33*. Following Prp22 depletion (in strain PADH1-701-TIR1) for the times indicated, samples were 4tU-labelled for 1 min. Data are normalized to conditions without depletion. (b) Occupancy of U2 snRNP-core component Lea1 was measured from depletion time course samples as in panel a. Solid red lines are Lea1 ChIP-qPCR for *ACT1* and *ECM33*, and dashed black lines are Prp22 levels measured by western blot (blot image not shown). ChIP data are normalized against background (intron-less *ALG9*). Prp22 western blot signal is normalized against internal control Pgk1. (c) RT-qPCR analysis of 4tU-labelled (1-min labelling) newly synthesized *ACT1* RNA, following Prp16 depletion in strain PZ4EV-NTIR1, with osTIR1 expression induced by β-estradiol[23]. Relative levels (%) of Prp16 protein are in X-axis below minutes. Data in panels (a) and (c) are normalized against exon 2. Data in all panels (a-c) are relative to no depletion (time 0) and x-axes represent time (minutes) after addition of auxin. Error bars denote standard error of four (panels a-b) or three (panel c) biological replicates.

**Figure 5.**
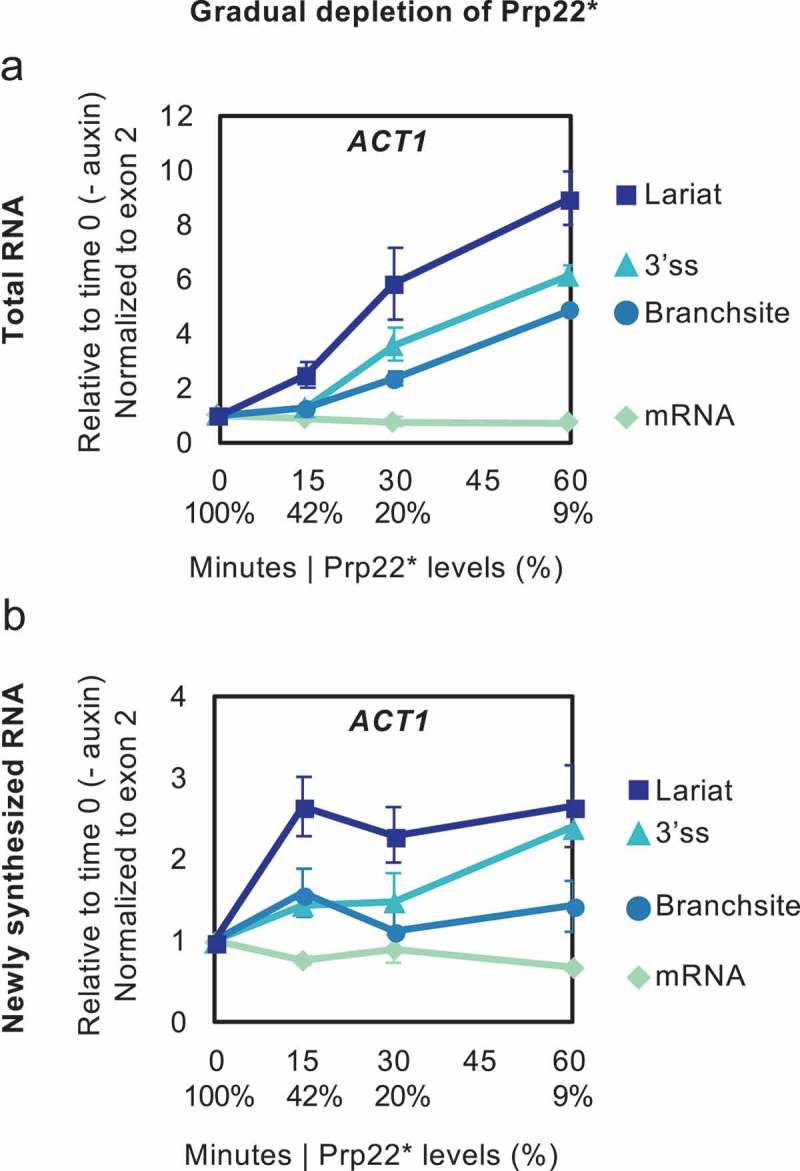
RNA Analysis in a time course of a more gradual Prp22 depletion. Prp22 was depleted in strain PADH1-409-TIR1, in which OsTIR1 is expressed to low levels[23] so the Prp22 level declines neither as far nor as fast as in the other figures. Splicing intermediates of *ACT1* were analysed by RT-qPCR from total RNA (a) and 4tU-labelled (labelled for 4 min at each time of depletion) newly synthesized RNA (b). Data are normalized to exon 2 and relative to no depletion (time 0). Relative levels (%) of Prp16 protein are in X-axis below minutes. Error bars denote standard error of biological triplicates.

See Table S1 for yeast strain genotypes. The OsTIR1 auxin-binding protein was expressed in *S. cerevisiae* strains PADH1-701-TIR1 or PADH1-409-TIR1 (depletes more gradually than PADH-701) directed by constitutive P*_ADH1_* promoters, whereas in PZ4EV-NTIR1 it is subject to regulated expression from a β-estradiol-inducible promoter [25] as previously described [23]. Target proteins were C-terminally tagged with AID*-6FLAG (referred to in the text simply as ‘AID-tagged’) [26], using a PCR‐based method to alter the coding sequence on the genome [27]. Prp16, Prp22 and Prp45 were individually depleted in PADH1-701-TIR1 while Prp4 was depleted in PADH1-409-TIR1 (Figs. 1–3). Depletion of Prp16 in Fig. 4c was done in strain PZ4EV-NTIR1. Where specified, more gradual depletion of Prp22 (Fig. 5) was performed in strain PADH1-409-TIR1. Yeast strains were grown at 30°C on Yeast Peptone Dextrose supplemented with adenine (YPDA). Protein depletions were performed as previously described [23].

### Antibodies used

Western blots were performed as previously described [23], using rat anti‐FLAG (Agilent, Cat. No. 200474) and mouse anti‐PGK1 (Abcam, Cat. No. Ab113687) antibodies. The anti-FLAG signal was normalized against Pgk1, which is encoded by an intronless gene and was used as internal control. Rabbit anti-Prp40 polyclonal antibodies (our laboratory) were used for ChIP of U1 snRNP, and mouse anti-HA 12CA5 monoclonal antibodies (Roche, Cat. No. 11583816001) were used for ChIP and RIP of U2 snRNP.

### RNA analysis and chromatin immunoprecipitation

Reverse transcription (RT) was performed as previously described [28]. 2x SYBR green III master mix (Agilent Cat. 600882-51) was used for quantitative polymerase chain reaction (qPCR). Oligonucleotide primers for RT and qPCR are listed in Supplemental Methods. RNA immunoprecipitation (RIP) was performed as previously described [29]. Protocol for chromatin immunoprecipitation-quantitative PCR (ChIP-qPCR) is described in Supplemental Methods. Isolation of total RNA and newly synthesized RNA (nsRNA) by 4-thio-uracil (4tU) labelling were performed as described previously [30] with a few modifications [31].

## Results

### Depletion of Prp16 or Prp22 causes a first step of splicing defect

We investigated the effect on splicing efficiency in vivo of depleting Prp4, Prp16, Prp22 or Prp45. For this we individually C-terminally tagged these proteins with the AID*-6FLAG tag [26] in *Saccharomyces cerevisiae* strains that constitutively express the plant auxin-binding receptor TIR1. We added auxin to cultures of the AID-tagged strains and took samples for analysis immediately (T0) and after 30-min incubation (T30). By western blot analysis, we estimated that Prp4 was depleted to around 20%, and Prp45, Prp16 and Prp22 were depleted to less than 7% of initial values (Fig. 1b). Having confirmed that targeted depletion was successful, we measured the relative abundance of the pre-mRNA, the lariat-exon2 splicing intermediate and spliced mRNA of *ACT1* transcripts by reverse transcriptase real-time quantitative PCR (RT-qPCR) using specific primers (Fig. 1c,d). As anticipated, depletion of tri-snRNP protein Prp4 or NTC-related Prp45 (proteins recruited before the first step of splicing) led to an increase in signal across both the branchsite (BS), representing unspliced pre-mRNA; and 3ʹ splice site (3’ss), due to unspliced pre-mRNA or lariat-exon2 levels, while lariat levels decreased due to reduced production of lariat-exon2 and/or excised intron. These observations indicate a defect in the first step of splicing. Depletion of Prp16 or Prp22 led to increased lariat and 3’ss signals, indicative of a second step defect but, unexpectedly, also to increased BS levels, suggesting that both steps of splicing were negatively affected by reduction in these late-acting splicing factors. Because it is unlikely that both Prp16 and Prp22 have an additional and uncharacterized role in the first catalytic step of splicing, we speculated that the observed phenotype was an indirect consequence of depleting these proteins.

### Pre-spliceosome formation is reduced in the absence of Prp4, Prp45, Prp16 or Prp22

It was previously demonstrated that the co-transcriptional recruitment of splicing factors can be monitored in vivo using the chromatin immunoprecipitation (ChIP) approach, because splicing factors bound to nascent transcripts are sufficiently close to RNA polymerase to interact, either directly or indirectly, with the DNA template [29,32–36]. Therefore, to study the effect on early stages of co-transcriptional spliceosome assembly of depleting Prp16 or Prp22, we performed ChIP of Prp40 and HA-tagged Lea1, as core components of U1 and U2 snRNP, respectively, at the well characterized intron-containing *ACT1* and *ECM33* genes. In the undepleted controls, we observe the typical profiles for ChIP of U1 and U2 snRNP components, with both signals high over exon 2 but U1 snRNP signal declining more 5ʹ than the U2 signal, representing release of the U1 snRNP as B complex forms (Fig. 2) [33,34]. Following depletion of Prp4, we observe lower occupancy of Prp40 (U1) and Lea1 (U2), compared to the undepleted control. Given that Prp4 joins after pre-spliceosome formation (Fig. 1a), these data suggest that depleting Prp4 also causes an unexpected defect in U1 and U2 snRNP recruitment. In the absence of Prp45, we observe lower occupancy of Lea1 (U2), and higher occupancy of Prp40 (U1) towards the 3ʹ end of the genes (Fig. 2), indicating reduced and possibly delayed U2 recruitment, which agrees with our previous observation of Prp45 depletion causing a first step of splicing defect. The elevated U1 signal is likely caused by failure to form B complex, which normally involves U1 release [37]. In the absence of either Prp16 or Prp22, we again observe lower occupancy of Lea1 (U2) and higher occupancy of Prp40 (U1), similar to depletion of Prp45. Given that each of these four factors is thought to function after U1 and U2 snRNP recruitment, the reduced ChIP signal for U2 snRNP suggests that depleting any one of these proteins also causes a defect at an earlier than expected stage of spliceosome assembly.

To explain these unusual observations, we hypothesize that depletion of any of these four splicing factors causes accumulation of arrested complexes, with splicing components becoming sequestered, which leads to reduced spliceosome assembly on newly synthesized transcripts and a first step splicing defect.

### Depletion of splicing factors correlates with increased snRNP interactions

To test the hypothesis that splicing complexes accumulate in cells depleted of these splicing factors, we performed an RNA immunoprecipitation (RIP) analysis where HA-tagged Lea1 (U2) was pulled down and the associated snRNAs were measured by RT-qPCR. The normal pattern of Lea1 (U2 snRNP) association with U1, U4, U5 and U6 snRNAs is presented in Fig. 3a. Prp4 is required for tri-snRNP recruitment, a step necessary for the transition of pre-spliceosome to pre-B complex (Fig. 1a) [38]. Therefore, based on the ChIP data we anticipate that absence of this protein may lead to the accumulation of pre-spliceosome complexes (U1 and U2 snRNPs). Indeed, we observe that depletion of Prp4 correlates with increased association of Lea1 with U1 snRNA, and decreased association with U4, U5 and U6 snRNAs, relative to the undepleted control (Fig. 3b).

In the case of Prp45 depletion, the slightly elevated association of Lea1 with U1 and U4 compared with the undepleted control may indicate accumulation of pre-B and/or B complex, as a result of inefficient conversion of B complex to Bact complex (Fig. 3b). Prp45 is an NTC-related protein, and it has been proposed that the NTC functions at the transition from B to Bact complex by stabilizing the association of the U5 and U6 snRNAs with the 5ʹ end of the intron after U4 is dissociated [39]. In view of the slightly reduced association of Lea1 with U5, ChIP was performed for the U5 snRNP protein, Prp8, showing that co-transcriptional recruitment of U5 snRNP was also reduced following depletion of Prp45 (Fig S1), adding further support for failure to form a stable B complex.

In contrast, depletion of Prp16 or Prp22, correlates with increased association of Lea1 with U5 and U6 snRNAs, suggesting accumulation of Bact and/or C complexes that contain U2, U6 and U5 snRNAs. In the case of Prp16 depletion, there is also decreased association with U1 and U4 snRNAs (Fig. 3b), possibly indicating reduced pre-spliceosome and/or B complex formation. These observations resemble reports of arrested spliceosome accumulation in temperature-sensitive *prp2, prp16* and *prp22* mutant strains [40]. As the ChIP results show reduced co-transcriptional recruitment of U2 snRNP (and likely also of U5 and U4/U6 snRNPs, whose assembly requires pre-spliceosome formation), we conclude that the RIP data reflect the post-transcriptional accumulation of stalled spliceosomes. This can explain both the reduced U2 recruitment to newly synthesized transcripts (Fig. 2) and pre-mRNA accumulation (Fig. 1d).

### Kinetic analysis of Prp22 and Prp16 depletion

Next, we studied the kinetics of the splicing defect caused by Prp22 depletion by analysing both 4-thiouracil (4tU)-labelled nascent RNA [30] and co-transcriptional recruitment of Lea1 (U2 snRNP) at short times (0, 3, 6 and 12 min) after auxin addition. Labelling RNA with 4tU in vivo for as little as 1 min allows the isolation of newly synthesized RNA (nsRNA), and its production and processing can be assessed as the protein is depleted, whereas total RNA (Fig. 1) includes RNA produced prior to target protein depletion. The 4tU splicing analysis shows *ACT1* lariat abundance elevated compared to the signal from exon 2 in the sample incubated with auxin for 3 min, then decreasing (relative to exon 2), as the BS (pre-mRNA) signal builds up (Fig. 4a). Moreover, the 3’ss signal also accumulates transiently in parallel with the lariat signal, levels off as lariat declines and BS increases, then accumulates again in parallel with the BS signal. These results indicate that the lariat-exon2 product of the first step of splicing accumulates transiently before a first step defect kicks in and prevents further lariat-exon2 production.

Consistently, the ChIP signal for Lea1 (U2 snRNP) at *ACT1*, is reduced in the 6-min sample but not in the 3-min sample (Fig. 4b), indicating reduced recruitment of U2 snRNP to nascent RNA after only 6 min of Prp22 depletion. This can explain the first step defect detected at 6 min as being due to reduced co-transcriptional spliceosome assembly. Taken together, the kinetic analyses indicate that although the first step splicing defect occurs extremely rapidly after auxin addition, it is preceded by a second step defect, arguing that the first step splicing defect is not a direct consequence of Prp22 depletion, but a secondary effect. In the case of *ECM33* transcript, we observe 5’ss (pre-mRNA) signal increase at 6 min, and also reduced recruitment of U2 snRNP (Lea1) already at 3 min (Fig. 4a, B; specific assay of *ECM33* lariat by RT-qPCR has not been achieved).

We performed a similar kinetic analysis of Prp16 depletion, this time using a Prp16 AID-tagged strain in which TIR1 is conditionally expressed by the addition of β-estradiol prior to auxin addition, permitting tighter control of the system [23]. The results were similar to those for Prp22 depletion, with lariat signal peaking at 6 min after auxin addition, then decreasing as the BS signal increased but, notably, after a significant lag (Fig. 4c).

### A more gradual depletion of Prp22 increases contrast between primary and secondary effects

As depletion of Prp22 in the PADH1-701-TIR1 strain affected splicing extremely quickly, and from a situation where this protein was already partially depleted without auxin, we analysed another AID strain, PADH1-409-TIR1, that expresses TIR1 at a lower level and, therefore, allows a more gradual depletion of Prp22 and from a starting point of no auxin-independent depletion [23]. Analysis of total RNA (Fig. 5a) showed that reduction of Prp22 to 42% of the initial level took 15 min as opposed to 3 min with the more rapidly depleting strain (shown in Fig. 4b), and caused only a low level of lariat accumulation. At 30 min, when Prp22 was reduced to 20%, there was accumulation of more lariat, as well some 3’ss signal and, to a lesser extent, BS signal, indicating a small amount of pre-mRNA. At 60 min, with only 9% Prp22 remaining, the signals for lariat, 3’ss and BS were all strongly elevated.

4tU-labelling analysis of the kinetics of splicing with gradually depleted Prp22 (Fig. 5b) also showed that 15 min after auxin addition, with 42% of Prp22 remaining, there was significant accumulation of only the lariat signal, indicative of excised intron lariat accumulation. At 30 min (Prp22 at 20%) 3’ss signal remained low, whereas at 60 min (Prp22 at 9%) the 3’ss signal accumulated at a similar rate to lariat signal, and there was very little BS (pre-mRNA) accumulation, indicating build-up of lariat-exon2. In summary, during a more gradual Prp22 depletion, excised intron lariat clearly accumulated before the lariat-exon2 product of the first step, and there was little pre-mRNA accumulation, indicating that, under these milder depletion conditions, the first step of splicing was hardly affected.

## Discussion

Our observation of pre-mRNA accumulation when depleting Prp16 or Prp22 resembles previous studies showing pre-mRNA accumulation at the restrictive temperature in strains with heat-sensitive mutants of *PRP16* [41,42] and *PRP22* [17,41–43]. A homozygous lethal mutation of the zebrafish orthologue of Prp22 (called Dhx8), also caused accumulation of unspliced pre-mRNA [44], indicating that this phenotype is not specific to yeast. Company et al. [43]speculated that in a heat-sensitive *prp22* strain unspliced pre-mRNA accumulated because defective spliceosomes were not recycled for new rounds of splicing, but this was not investigated further. Evidence supporting the concept of defective spliceosomes limiting an earlier step came subsequently, in a report that metabolic depletion of U5 snRNA caused accumulation of arrested pre-spliceosomes and reduced co-transcriptional recruitment of U1 and U2 snRNPs [29], which is similar to our Prp4 depletion analysis. However, as the *GAL* promoter-driven expression of U5 snRNA was repressed for 16 h, it cannot be ruled out that the recycling defect was an indirect consequence of the prolonged splicing defect. For example, reduced expression of intron-containing genes that encode splicing factors (e.g. U1 snRNP protein Mud1) could explain the reduced co-transcriptional recruitment of U1 snRNP that was observed.

Our AID depletion approach is novel because (1) we target not just one, but different stages of splicing, (2) we deplete the target proteins very rapidly, (3) we analyse both the efficiency of splicing newly synthesized transcripts and co-transcriptional snRNP recruitment, and (4) we demonstrate the accumulation of different splicing complexes depending on the stage at which the depleted factor functions. Taken together, data derived from this approach and from previous studies [29,40] demonstrate that perturbing splicing can lead to a recycling defect that limits the earliest steps of the splicing cycle. We show that this ‘feed-back’ effect happens extremely rapidly, suggesting that spliceosome disassembly can become a rate-limiting step for splicing, and supporting the proposal that pre-mRNA substrates compete for a limited pool of spliceosome components in yeast [45]. Moreover, we demonstrate that the effect is indeed titratable; partial and/or slower depletion of Prp22 resulted in a less severe defect, allowing the kinetics of this effect to be analysed. Therefore, we propose that by controlling the rate and optimizing the extent of depletion, one can minimize secondary effects associated with recycling defects and thereby facilitate the functional analysis of a targeted protein.

Similar to our results for Prp45 depletion, Hálová et al. [46] also observed a reduction in co-transcriptional U2 snRNP recruitment when they analysed the effect of truncating the C-terminal domain of Prp45, proposing that Prp45 has an earlier than expected role in splicing, at the stage of U2 snRNP recruitment. Our observation that Prp45 depletion leads to enhanced U1 snRNP and reduced U2 snRNP signal at the site of transcription could support either a role for Prp45 in U2 snRNP recruitment or, alternatively, may indicate a recycling defect. Prp45 depletion also resulted in a mildly increased association of U2 with U1 and U4 (our RIP analysis), possibly indicating pre-B and/or B complex accumulation (Fig. 3b). This seems plausible, as Prp45 is an NTC-related protein, and it has been proposed that the NTC functions at the transition from B to Bact complex by stabilizing the association of the U5 and U6 snRNAs with the 5ʹ end of the intron after U4 is dissociated [39]. Prp45 has an extended conformation in the spliceosome [12], interacting with many spliceosome components. It is conceivable that depletion of Prp45 could directly affect more than one step in the splicing cycle, perhaps even in a substrate-specific manner, which could make mutation or depletion results difficult to interpret.

Chen et al. [47] tested the ability of the NTR disassembly complex (containing Prp43, Ntr1 and Ntr2) to mediate substrate release from the spliceosome when various DEAH-box RNA-stimulated ATPases were mutated or immunodepleted in vitro. They concluded that only spliceosome intermediate complexes that were arrested after, but not before, the ATP-dependent action of Prp2, Prp16 or Prp22 could be actively disassembled. Our in vivo analyses of Prp16 and Prp22 depletion are also consistent with failure to disassemble complexes stalled before these proteins act. Furthermore, our analyses show that recycling defects are not limited to knockdown of RNA-stimulated ATPases, as depletion of Prp4 or Prp3, another tri-snRNP protein, displayed similar defects (Fig. S2).

The DEAD/H-box RNA-stimulated ATPases were proposed to function as proofreading or fidelity factors in splicing based on assays using reporter genes encoding aberrant or suboptimal pre-mRNA substrates (e.g. pre-mRNAs with non-canonical splice sites or branchsites) [48,49]. It was further proposed that defective complexes are removed by a discard pathway [50,51]. In this work, we focused on a different type of aberrant spliceosomes – those that contain normal pre-mRNA substrates but defective splicing machinery. This situation leads to a wide-spread defect, that may overwhelm surveillance pathways, such that the splicing factors become sequestered in defective complexes, limiting the assembly of new spliceosomes. Interestingly, in the strain where Prp22 was more gradually depleted, accumulation of pre-mRNA (Branchsite) was both slower than lariat accumulation in the same conditions (Fig. 5a) and reduced compared to fast depletion of Prp22 (Fig. 1d), suggesting that a recycling defect is more likely when the target is rapidly depleted, and/or knocked down to low levels. Considering all evidence, it seems that the splicing surveillance machinery likely has the capability to recognize aberrant spliceosomes that either lack an essential splicing factor or contain a mutant protein. This notion is supported by evidence that mutations in disassembly factors Spp382 (Ntr1) and Prp43 suppress the defects caused by the spliceosome assembly mutations *prp38-1* and *prp8-1*, presumably by reducing dissociation of the defective complexes, thereby allowing more time for the defective splicing factors to function [52]. However, our results suggest that the splicing surveillance and/or discard machinery is not equipped to deal with a large or rapid accumulation of aberrant spliceosomes. We propose that when the splicing defect is highly penetrant, the surveillance capacity of the cell can be overwhelmed, leading to the build-up of arrested complexes. We speculate that this might also help to explain why some disease-causing splicing factor alleles in humans are more penetrant than others.

Our observed rapid accumulation of excised lariat upon Prp22 depletion (Fig. 5) is consistent with Prp22’s role in releasing spliced mRNA from the post-spliceosome [17–19], a step that ultimately leads to spliceosome disassembly by the NTR complex and recycling of the components. The high level of lariat and 3’ss signals but low level of branchsite signal for *ACT1* transcripts (indicative of a second step of splicing defect) after 3 min of rapid depletion (Fig. 4a) or 60 min of gradual depletion (Fig. 5b) of Prp22 in the 4tU-labelling analyses supports Prp22 having an additional role in the second catalytic step of splicing [20]. However, as excised lariat accumulated prior to lariat-exon2 (Fig. 5) it suggests that, in vivo, Prp22’s role in the second step of splicing is secondary to its role in mRNA release. An alternative explanation could be that accumulation of post-splicing complex following Prp22 depletion reduces the availability of second step factors such as Prp18 and Slu7 to below a limiting amount, thereby affecting the second step indirectly. Both Schwer & Gross [20] and our analyses of Prp22’s role in splicing were conducted only on *ACT1* transcripts. Therefore, it remains an open question whether this protein is also required for the second step of splicing of other transcripts.

Different intron-containing transcripts may be more or less sensitive to the depletion of different factors, and splicing may be affected at different stages. It is not always possible to distinguish first and second step splicing defects by RT-qPCR, as distinguishing unspliced pre-mRNA and lariat-exon2 intermediate species (as we have done for *ACT1*) generally depends on the specific measurement of lariats, which can be problematic. However, techniques have been developed to simultaneously sequence lariats of potentially all intron-containing transcripts of *S. cerevisiae* [53–55]. In principle, these transcriptome-wide approaches could be used to investigate, for example, whether Prp22 is required in vivo for the second step of splicing of only a subset of introns as has been proposed [20].

Our observations highlight a concern that, in order to avoid misleading conclusions when knocking down components of large complexes, it is important to consider possible indirect effects that can occur as a consequence of the accumulation of a disabled complex. This is a particular concern if the complex assembles and/or functions in a step-wise manner, such as the spliceosome or the ribosome assembly pathway. Ways of controlling for this include performing kinetic analyses of the effects, comparing the effects of depleting the target factor to different extents and at different rates, or of depleting different components of the complex. Therefore, when interpreting the results of large scale knock-down studies, for example, RNAi depletion analyses of human splicing factors [56–58], the possibility of confounding secondary effects should be considered.
