# Supplementary material for: Blocking late stages of splicing quickly limits pre-spliceosome assembly in vivo
Source: RNA Biol. 2019 Sep 4;16(12):1775–84. doi: 10.1080/15476286.2019.1657788 (PMC6844569; doi:10.1080/15476286.2019.1657788)
Supplement: Supplemental Material [file krnb-16-12-1657788-s001.zip › Supplementary information/Supplemental_figures_Mendoza-Ochoa et al_RNA Biology_3Aug.pdf]

SUPPLEMENTAL FIGURES

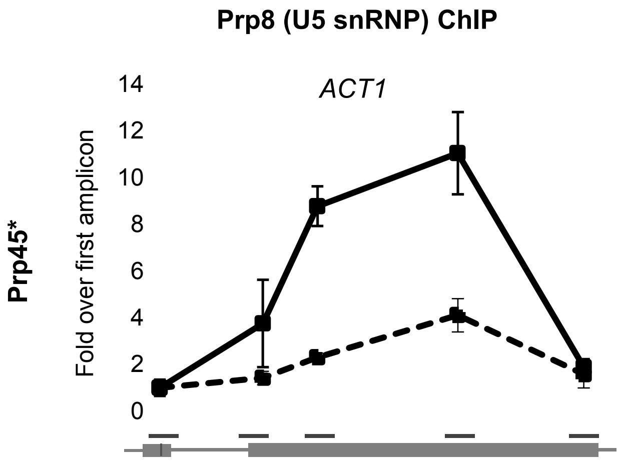

FIGURE S1. Depletion of Prp45 (as in Fig. 1) leads to reduced co-transcriptional recruitment of Prp8 (U5 snRNP) on *ACT1* gene. Antibodies used were rabbit anti-Prp8 polyclonal (our laboratory). Solid lines show Prp8 ChIP before depletion and dashed lines after depletion. The x-axis represents amplicon location within the gene. ChIP data are presented as relative to the first amplicon (exon 1). Error bars denote standard error of biological duplicates. * = AID*-6FLAG C-terminal tag.

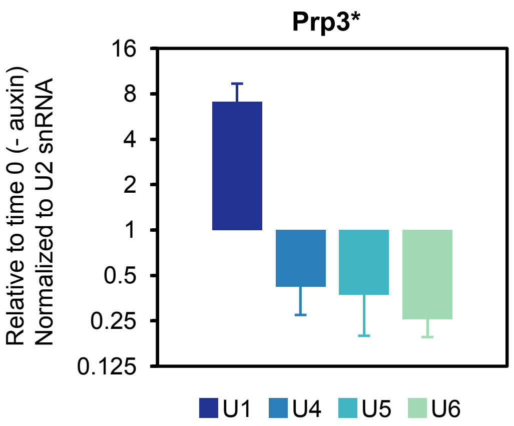

FIGURE S2. RIP analysis suggesting accumulation of arrested pre-spliceosome when depleting Prp3* (related to Figure 3B). RT-qPCR measurement of snRNAs associated with immunoprecipitated Lea1, core component of U2 snRNP, after depletion of tri-snRNP protein Prp3. Data are normalized to U2 snRNA signal and presented as relative to no depletion (time 0). Error bars denote standard error of biological triplicates. * = AID*-6FLAG C-terminal tag.
