# Supplementary material for: Blocking late stages of splicing quickly limits pre-spliceosome assembly in vivo
Source: RNA Biol. 2019 Sep 4;16(12):1775–84. doi: 10.1080/15476286.2019.1657788 (PMC6844569; doi:10.1080/15476286.2019.1657788)

Mendoza-Ochoa et al.

SUPPLEMENTAL METHODS AND MATERIALS

Chromatin immunoprecipitation

This method is based on “Yeast Chromatin Immunoprecipitation (ChIP) Protocol: Mechanical Breakage & FA Lysis Buffer” from the Meluh Lab, which can be found in: https://www.hopkinsmedicine.org/institute_basic_biomedical_sciences/research_centers/high_throughput_biology_hit/technology_center_networks_pathways/pdfs/protocols/MELUH_Yeast_ChIP_Protocol.pdf

1. Add 50 mL of log phase culture to a 50 mL falcon tube containing 1.37 mL of 37% formaldehyde and mix gently for 10 min.
2. Add 2.5 mL of 2.5 M glycine and mix gently for 5 min.
3. Recover the cell pellet by centrifugation and wash with 50 mL of ice-cold PBS. Wash with 1 mL ice-cold PBS while transferring the cell pellet to a 2 mL screw cap tube.
4. Add 300 µL of 0.5 mm zirconia beads and 350 µL of FA1 buffer.
5. Lyse the cells by shaking three times at 2,000 rpm in a Mini-Beadbeater-24 (BioSpec Products) for 2 min each time, with 2 min on ice in between.
6. Place the barrel of a 5 mL syringe inside a 15 mL falcon tube. To separate the lysate from the zirconia beads, with a flame-heated needle perforate the bottom of the 2 mL screw cap tube containing the sample, and place the tube inside the syringe barrel.
7. Spin this device for 1 min at 1000 x g. Transfer all the lysate to a new 1.5 mL tube.
8. To recover the chromatin fraction, spin the lysate at maximum speed for 15 min at 4ºC and then discard the supernatant.
9. Resuspend the pellet in 300 µL of FA1 buffer, transfer sample to a Diogenode tube, and sonicate for 10 cycles of 30 sec ON and 30 sec OFF at 4ºC in a Bioruptor Pico water bath-sonicator (Diagenode).
10. To recover the solubilized chromatin fraction, spin for 5 min at maximum speed at 4ºC, and transfer the supernatant to a 1.5 mL tube. Discard the pellet.
11. Quantify total proteins by Bradford.
12. To immunoprecipitate, mix 20 µL of dynabeads (coupled to protein A or G), 5 µg of antibody and 500 µg of chromatin extract, and incubate overnight at 4ºC in a rotating wheel.
13. Using a magnetic rack and a vacuum aspirator, wash the beads three times with FA1, three times with FA2, three times with FA3 and once with 0.05% Tween-20/TBS buffer (during the final wash of each buffer, mix by inversion until beads have resuspended completely and then spin for 10 sec at 100 g before the next step)
14. During the final washing step, transfer the beads to a new tube.
15. To elute the DNA and reverse cross-link, add 150 µL of ChIP elution buffer and 75 µg of Proteinase K to the washed beads and, as a separate sample, to 50 µg of chromatin extract as the 10% input control.
16. Incubate the samples at 42ºC for 2 hours, and 4 hours at 65ºC, while shaking at 1400 rpm.

17. Separate the supernatant from the beads, discard the beads, and purify the DNA using the Qiagen MinElute PCR kit. Take the purified DNA to a final volume of 400 µL with ddH2O.
18. Analyse by qPCR.

Table S1: Buffers

**Name**	**Component**	**Volume**
FA1	HEPES KOH 0.5 M pH 7.5	25 mL
	NaCl 4M	8.75 mL
	EDTA 0.5M pH8	0.5 mL
	TRITON	2.5 mL
	Na deoxycholate 10%	2.5 mL
	cOmplete Prot. Inhibitor*	1 tab/50 mL
	ddH20	to 250 mL
FA2	HEPES KOH 0.5 M pH 7.5	25 mL
	NaCl 4M	31.25 mL
	EDTA 0.5M pH8	0.5 mL
	TRITON	2.5 mL
	Na deoxycholate 10%	2.5 mL
	ddH20	to 250 mL
FA3	Tris-HCl pH8 1M	2.5 mL
	LiCl 4M	15.6 mL
	EDTA 0.5M pH8	0.5 mL
	NP-40	1.25 mL
	Na deoxycholate 10%	12.5 mL
	ddH20	to 250 mL
ChIP elution buffer	5 ml TRIS-HCl pH 7.5 1M	5 mL
	1.75 ml EDTA 0.5mM	1.75 mL
	SDS 10%	5 mL
	ddH20	to 50 mL

* dissolve cOmplete tablet in working solution and use immediately

Table S2: Primers for RT-qPCR

Lariat	ACT_BL_F	AGGGGCTTGAAATTTGGAAAAA
	**ACT_L_R**	GCAAGCGCTAGAACATACATAGTACA
Branchsite	ACT_BL_F	AGGGGCTTGAAATTTGGAAAAA
	**ACT_B_R**	GCAACAAAAAGAATGAAGCAATCG
3'ss	ACT_3_F	TTGCTTCATTCTTTTTGTTGCT
	**ACT_3m_R**	GCAAAACCGGCTTTACACAT
mRNA	ACT_m_F	TCGAAAATTTACTGAATTAACAATGGA
	**ACT_3m_R**	GCAAAACCGGCTTTACACAT
Exon 2	ACT_E_F	GCTGCTTTGGTTATTGATAACGGTTC
	**ACT_E_R**	GATGGGAAGACAGCACGAGGAG
5'ss	ECM33_V_F	AGTGCCTCCGCTCTAGCTGGT
	**ECM33_V_R**	CGAGATTTGTGAGGAAAGAGGCAAA
mRNA	ECM33_m_F	GCCTCCGCTCTAGCTGCTAACTC
	ECM33_m_R	TTGAGCAGTAGCAGTGGCAGAAGT
Exon 2	ECM33_E_F	CTTCTGCCACTGCTACTGCTCAAG
	**ECM33_E_R**	AGCAGCGGAACCCAAGTCAC

N.B. Primers in bold are added to the RT mix

Table S3: Primers for ChIP-qPCR

1	Act1_-71_F	TACATCAGCTTTTAGATTTTTCACGCTTACTGCTT
	Act1_+34_R	GATGGTGCAAGCGCTAGAACATACCAGAAT
2	Act1_+368_F	TGTACTAACATCGATTGCTTCATTCTTTTTGTTGC
	Act1_+413_R	GACGATAGATGGGAAGACAGCACGAGGA
3	Act1_+561_F	ATCTGGCATCATACCTTCTACAACGA
	Act1_+653_R	GTTTGATTTAGGGTTCATTGGAGCTT
4	Act1_+1119_F	TCTGCCGGTATTGACCAAACTACTTA
	Act1_+1210_R	CCGGACATAACGATGTTACCGTATAA
5	Act1_+1595_F	ATGTGTTTTGTCTCTCCCTTTTCTACGAAAATTTC
	Act1_+1734_R	TGATCATATGATACACGGTCCAATGGATAAACAT
1	ECM33_-592_F	GCAGTATCATCCTTCACGACCC
	ECM33_-510_R	GCGTCTTTCCCGTTTTTGC
2	ECM33_+9_F	CAAGAACGCTTTGACTGCTACTG
	ECM33_+145_R	GAAGAGGACCACGAATCTACTCG
3	ECM33_+430_F	ACTTCTGCCACTGCTACTGCTC
	ECM33_+562_R	AGGAACCATCAATCTCTTGGATAC
4	ECM33_+1073_F	TTGGTCAATCTTTGTCTATCGTCTC
	ECM33_+1173_R	TGTGTTGTTAGCAATGATGAAACC
5	ECM33_+1531_F	TCTAAGAAGTCTAAGGGTGCTGCTC
	ECM33_+1582_R	TGAATGAAGTGGCTGGAACAAG

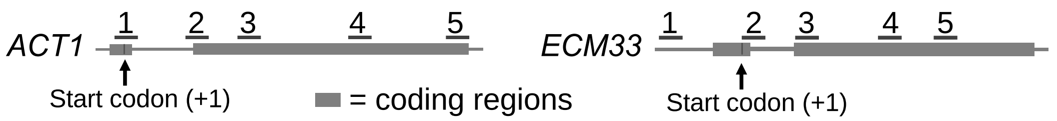

Supplement: Supplemental Material [file krnb-16-12-1657788-s001.zip › Supplementary information/Supplemental_methods_Mendoza-Ochoa et al_RNA Biology_3Aug.pdf]
