# Supplementary material for: Blocking late stages of splicing quickly limits pre-spliceosome assembly in vivo
Source: RNA Biol. 2019 Sep 4;16(12):1775–84. doi: 10.1080/15476286.2019.1657788 (PMC6844569; doi:10.1080/15476286.2019.1657788)
Supplement: Supplemental Material [file krnb-16-12-1657788-s001.zip › Supplementary information/Supplemental_strains_Mendoza-Ochoa et al_RNA Biology_3Aug.pdf]

Table S1 Mendoza-Ochoa et al. ,

Strain	Genotype	Parental strain	Figure	Source
PADH1-701-TIR1	MATα *ade2-1 his3-11,15 trp1-1 leu2-3,112 can1-100 ura3-1*::P*ADH1*-701-Os*TIR1-URA3*	-	-	Mendoza-Ochoa et al., 2018
PADH1-409-TIR1	MATα *ade2-1 ura3-1 trp1-1 leu2-3,112 can1-100 his3-11,15::*P*ADH1*-409-Os*TIR1-NatMX*	-	-	Mendoza-Ochoa et al., 2018
PZ4EV-NTIR1	MATα *ura3*Δ *leu2*Δ0::P*ACT1*-Z4EV-NatMX *ape2*::KanMX-Z4EVpr-NLS-Os*TIR1*-V5	-	-	Mendoza-Ochoa et al., 2018
Prp22-AID*-6FLAG_PADH1-701-TIR1	MATα *ade2-1 his3-11,15 trp1-1 leu2-3,112 can1-100 ura3-1*::P*ADH1*-701-Os*TIR1-URA3* *PRP22::PRP22*-AID*-6FLAG-HygMX	PADH1-701-TIR1	1B,D	Mendoza-Ochoa et al., 2018
Prp22-AID*-6FLAG_pFUI_PADH1-409-TIR1	MATα *ade2-1 ura3-1 trp1-1 leu2-3,112 can1-100 his3-11,15::*P*ADH1*-409-Os*TIR1-NatMX* *PRP22::PRP22*-AID*-6FLAG-HygMX [pRS426-FUI1-URA3]	PADH1-409-TIR1	5	Mendoza-Ochoa et al., 2018
Prp16-AID*-6FLAG_PADH1-701-TIR1	MATα *ade2-1 his3-11,15 trp1-1 leu2-3,112 can1-100 ura3-1*::P*ADH1*-701-Os*TIR1-URA3* *PRP16::PRP16*-AID*-6FLAG-HygMX	PADH1-701-TIR1	1B,D	This study
Prp45-AID*-6FLAG_PADH1-701-TIR1	MATα *ade2-1 his3-11,15 trp1-1 leu2-3,112 can1-100 ura3-1*::P*ADH1*-701-Os*TIR1-URA3* *PRP45::PRP45*-AID*-6FLAG-HygMX	PADH1-701-TIR1	1B,D	This study
Prp4-AID*-6FLAG_PADH1-409-TIR1	MATα *ade2-1 ura3-1 trp1-1 leu2-3,112 can1-100 his3-11,15::*P*ADH1*-409-Os*TIR1-NatMX* *PRP4::PRP4*-AID*-6FLAG-HygMX	PADH1-409-TIR1	1B,D	This study
Prp3-AID*-6FLAG_PADH1-409-TIR1	MATα *ade2-1 ura3-1 trp1-1 leu2-3,112 can1-100 his3-11,15::*P*ADH1*-409-Os*TIR1-NatMX* *PRP3::PRP3*-AID*-6FLAG-HygMX	PADH1-409-TIR1	S2	This lab (Barbara Terlouw)
Prp22-AID*-6FLAG_Lea1-3HA_PADH1-701-TIR1	MATα *ade2-1 his3-11,15 trp1-1 leu2-3,112 can1-100 ura3-1*::P*ADH1*-701-Os*TIR1-URA3* *PRP22::PRP22*-AID*-6FLAG-HygMX *LEA1::LEA1*-3HA-*HIS3MX6*	Prp22-AID*-6FLAG_PADH1-701-TIR1	2-3	This study
Prp16-AID*-6FLAG_Lea1-3HA_PADH1-701-TIR1	MATα *ade2-1 his3-11,15 trp1-1 leu2-3,112 can1-100 ura3-1*::P*ADH1*-701-Os*TIR1-URA3* *PRP16::PRP16*-AID*-6FLAG-HygMX *LEA1::LEA1*-3HA-*HIS3MX6*	Prp16-AID*-6FLAG_PADH1-701-TIR1	2-3	This study
Prp45-AID*-6FLAG_Lea1-3HA_PADH1-701-TIR1	MATα *ade2-1 his3-11,15 trp1-1 leu2-3,112 can1-100 ura3-1*::P*ADH1*-701-Os*TIR1-URA3* *PRP45::PRP45*-AID*-6FLAG-HygMX *LEA1::LEA1*-3HA-*HIS3MX6*	Prp45-AID*-6FLAG_PADH1-701-TIR1	2-3	This study
Prp4-AID*-6FLAG_Lea1-3HA _PADH1-409-TIR1	MATα *ade2-1 ura3-1 trp1-1 leu2-3,112 can1-100 his3-11,15::*P*ADH1*-409-Os*TIR1-NatMX* *PRP4::PRP4*-AID*-6FLAG-HygMX *LEA1::LEA1*-3HA-*HIS3MX6*	Prp4-AID*-6FLAG_PADH1-409-TIR1	2-3	This study
Prp22-AID*-6FLAG_Lea1-3HA_pFUI_PADH1-701-TIR1	MATα *ade2-1 his3-11,15 trp1-1 leu2-3,112 can1-100 ura3-1*::P*ADH1*-701-Os*TIR1-URA3* *PRP22::PRP22*-AID*-6FLAG-HygMX *LEA1::LEA1*-3HA-*HIS3MX6* [pRS425-FUI1-LEU2]	Prp22-AID*-6FLAG_Lea1-3HA_PADH1-701-TIR1	4	This study
Prp16-AID*-6FLAG_pFUI_PZ4EV-NTIR1	MATα *ura3*Δ *leu2*Δ0::P*ACT1*-Z4EV-NatMX *ape2*::KanMX-Z4EVpr-NLS-Os*TIR1*-V5 *PRP16*::*PRP16*-AID*-6FLAG-HygMX [pRS426-FUI1-URA3]	PZ4EV-NTIR1	4	This study

Mendoza-Ochoa GI, Barrass JD, Terlouw BR, Maudlin IE, de Lucas S, Sani E, Aslanzadeh V, Reid JAE, Beggs JD. A fast and tuneable auxin-inducible degron for depletion of target proteins in budding yeast. Yeast 2018; :1–7. Available from: http://doi.wiley.com/10.1002/yea.3362

Where indicated, strains were transformed with yeast uracil permease FUI1 overexpressed on 2 μM plasmids (ref. 30), either pRS426 (URA3 marker) or pRS425 (LEU2 marker), permitting 4tU-labelling of nascent RNA
